# Recent advances in rectal cancer treatment – are we on the right track?

**DOI:** 10.48101/ujms.v129.10537

**Published:** 2024-02-21

**Authors:** Bengt Glimelius

**Affiliations:** Department of Immunology, Genetics and Pathology, Uppsala University, Uppsala, Sweden

**Keywords:** Locally advanced rectal cancer, organ preservation, watch and wait, total neoadjuvant treatment

## Abstract

**Background:**

Staging and treatment of rectal cancer have evolved over several decades with considerably fewer locoregional recurrences but no marked improved survival since systemic recurrence risks remain virtually unchanged. This development will briefly be summarised followed by a thorough discussion of two recent developments.

**Methods:**

A systematic approach towards the literature is aimed at focusing on organ preservation and the delivery of all non-surgical treatments prior to surgery or total neoadjuvant treatment (TNT).

**Results:**

Organ preservation, that is to defer surgery if the tumour happens to disappear completely after any pre-treatment given to locally advanced tumours to decrease recurrence risks has increased in popularity and is, if not universally, widely accepted. To give neo-adjuvant treatment to intentionally obtain a clinically complete remission to avoid surgery is practised in some environments but is mostly still experimental. TNT, that is to provide both radiotherapy and chemotherapy aimed at killing microscopic disease in the pelvis or elsewhere has been subject to several trials. Collectively, they show that the chance of achieving a complete response, pathologically or clinically, has approximately doubled, increasing the chance for organ preservation, and the risk of distant metastasis has decreased at least in some trials. The best schedule remains to be established.

**Conclusions:**

To obtain substantial progress and also improve survival, the systemic treatments need to be improved even if preoperative delivery is more effective and better tolerated than postoperative. The locoregional treatment may be further optimised through better risk prediction.

## Introduction

Over several decades, advances have been seen in the treatment of rectal cancer resulting in a marked reduction in the risk of locoregional recurrences (LRRs), but without any improved overall survival (OS) in the trials (1). Population data have, however, revealed improved OS during the past decades (2, 3). The LRRs are usually disabling, difficult to treat and have a poor prognosis (4), and it is therefore legitimate to decrease them even if OS is not improved. The advances are reached, thanks to better possibilities to stage the tumours, using magnetic resonance imaging (MRI) allowing better selection to different treatments, an increased use of preoperative radiotherapy alone or together with chemotherapy (RT/CRT) and more precise surgery with dissection in the embryonic plane outside the mesorectal fascia (MRF, total mesorectal excision, TME).

**Figure 1:**
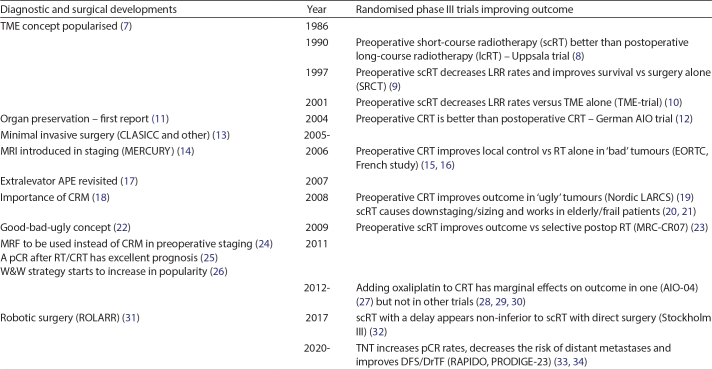
Progress in rectal cancer treatment during recent decades. Figure 1. Randomised trials of radiotherapy (RT) or chemoradiotherapy (CRT) prior to surgery having improved outcome are shown in the right column by year of the main publication. In the left column, important diagnostic and surgical improvements are shown by the year of the most relevant, not necessarily the first publication. TME, total mesorectal excision, APE abdomino-perineal excision, CRT, chemoradiotherapy using a fluoropyrimidine, MRI, magnetic resonance imaging, CRM, circumferential resection margin, MRF, mesorectal fascia, scRT, short-course radiotherapy (5 Gy × 5), lcRT, long-course radiotherapy (1.8–2Gy × 25–28), LRR, locoregional recurrence. DFS, disease-free survival, DrTF, disease-related treatment failure, TNT, total neoadjuvant treatment, W&W, watch and wait. This review focuses on the latter two developments, W&W and TNT.

This review will briefly describe the knowledge behind the treatments about a decade ago (reviewed in this journal in (5, 6) and then, in greater detail, describe and discuss two of the most recent advances. Major reasons behind the progress seen during recent decades are illustrated in Figure 1.

## Knowledge base behind rectal cancer treatment about 10 years ago or around 2014

Several randomised trials had established that preoperative RT/CRT, being more effective than postoperative RT/CRT (8, 12), decreases the risk of LRR by over half irrespective of whether surgery was ‘old-fashioned’ or ‘more precise’, that is TME (9, 10, 23). OS was improved when the absolute risk after surgery alone was high or above 20% as it was after ‘old-fashioned’ surgery (9, 35), but not after TME when the absolute risk was about 10% (10, 23, 36). Applying a ‘good-bad-ugly’ or ‘early-intermediate-locally advanced’ concept (22), it was established that in ugly/locally advanced tumours, where downsizing or downstaging of the tumour is required, the addition of chemotherapy to long-course RT (lcRT) (about 45–50 Gy for 5–5½ weeks, i.e. CRT) improved local control, but not OS (15, 16, 19). In less advanced or bad/intermediate risk tumours, where the tumour was judged resectable upfront and no downsizing/staging was required, both short-course RT (5 × 5 Gy in 1 week, scRT) or long-course CRT reduced LRR-rates to low levels, and both alternatives were recommended in guidelines. Even if much less demanding, scRT was not universally accepted above CRT even when at least two trials could not show any difference in tumour outcome (37, 38). Not only financial issues but also unfounded concerns about late toxicity after scRT were behind these different opinions.

Since no downsizing/staging was required in intermediate risk tumours, the scRT schedule was immediately followed by surgery or preferably within 10 days after the first radiation fraction (39). It was soon realised that if the tumour was then non-resectable, surgery several weeks later could be successful, and, in some patients, the tumour was entirely gone, that is a pathological complete remission (pCR) was seen. A delay in surgery was also more and more practised in elderly and/or fail patients not tolerating the reference treatment CRT (20, 21). The Stockholm III trial explored the value of delaying surgery after scRT and confirmed the downstaging effect of the delay. The delay was tolerable in most patients (40), and it resulted in less surgical complications and had apparently similar oncological outcome (32). The trial also included a third arm, lcRT without any chemotherapy with no advantage over scRT.

Despite favourable effects on recurrence rates and survival in colon cancer, adjuvant chemotherapy in rectal cancer did not sufficiently improve outcomes. A Cochrane analysis had indicated some improvements mainly in patients operated directly (41), but two meta-analyses did not reveal any significant gains in patients treated with RT/CRT before surgery (42, 43). Since then, no randomised trials with a postoperative surgery alone arm have been reported. Multiple retrospective studies have been performed including numerous reviews trying to explain why it does not work (sufficiently) [e.g. (44)]. In one of the randomised trials exploring the value of total neo-adjuvant therapy (TNT, the RAPIDO trial, to be described below), adjuvant chemotherapy was optional in the standard arm providing CRT preoperatively, reflecting the different opinions worldwide. Propensity score stratification suggested that adjuvant chemotherapy reduced recurrence risks and improved disease-free survival (DFS) by 20–25% or in the same order of magnitude as indicated by the randomised trials/meta-analyses, however, not statistically significantly (45).

### What is a rectal cancer and what is a locally advanced rectal cancer?

The rectum is the most distal part of the large bowel, but the delineation of the border between colon and rectum has been variably interpreted, and many definitions exist. An international consensus group suggested that the sigmoid take-off as seen on CT or MRI should define the border (46). Although this point is easily visible on a lateral imaging view, it is not available when a doctor has identified a new bowel malignancy and needs to decide whether staging should be as for a rectal cancer, always including MRI, or as a colon cancer where MRI is not part of the routine. In many countries and in many trials, the distal part of the tumour should be below a defined number of cm (e.g. 15 cm) above the anal verge, preferably measured with a rigid rectoscope, to be classified as rectal.

The most widely used definition of what is considered locally advanced (LARC) is any tumour staged as clinical stage cT3–4 or N+. Using this definition, about 80% of newly diagnosed rectal cancers are locally advanced (47). This definition is still extensively used (48) but means that far too many patients will receive intensive treatment resulting in unnecessary toxicity. The ‘good-bad-ugly’ concept identifies an intermediate group composed of the less advanced cT3–4 or N+ tumours. The distinction between the bad and ugly groups varies between guidelines and trials. It is usually based upon the risk of LRR and not primarily upon the risk of distant metastases. Most cT4s are referred to the ugly group as are those with MRF-involvement and signs of lateral node involvement. Staging of the mesorectal lymph nodes is difficult with mainly over-staging but also under-staging since small, homogenous, and regular nodes can contain metastatic deposits. A few guidelines do not consider nodal stage at all, whereas others do (48). Rather than trying to evaluate the nodes on MRI, some state you could just as well ‘flip a coin’ (49–51). The most recent Swedish guidelines are shown in Table 1. According to these guidelines, nodal status is less important than in the past but not ignored as, for example in Norway or Denmark (48).

**Table 1:** Indications for preoperative treatment in rectal cancer according to MRI characteristics. Swedish national care programme from 2020 (52).

align="left">cTN, level above anal verge	align="center">T1-2	align="center">T3ab	align="center">T3cd	align="center">T4a^a^	align="center">T4b, easy	align="center">T4b difficult	align="center">N0-1	align="center">N2^c^	align="center">EMVI+	align="center">MRF+ primary^b^	align="center">MRF+ TD or nodes	align="center">Lateral nodes
High 10–15 cm entirely above peritoneal reflection	0	0	0	5 × 5	0	CRT/TNT	0	0	0	--	--	--
High 10–15 cm	0	0	0	0	0	CRT/TNT	0	0	5 × 5	CRT/TNT	5 × 5	CRT/TNT
Middle 5–10 cm	0	0	5 × 5	5 × 5	5 × 5	CRT/TNT	0	5 × 5	5 × 5	CRT/TNT	5 × 5	CRT/TNT
Low 0–5 cm above intersphincteric plane	0	0	5 × 5	--	5 × 5	CRT/TNT	0	5 × 5	5 × 5	CRT/TNT	5 × 5	CRT/TNT
Low 0–5 cm in the intersphincteric plane	5 × 5	5 × 5	5 × 5	–	5 × 5	CRT/TNT	0	5 × 5	5 × 5	CRT/TNT	5 × 5	CRT/TNT

0 means that the tumour is ‘early or good’, and surgery alone is recommended; 5 × 5 means that the tumour is ‘intermediate’ or ‘bad’, and scRT alone with immediate or delayed surgery is recommended, and no down-staging/sizing needed; CRT/TNT means that it is an advanced tumour or ‘ugly’ with a need for down-staging/sizing. CRT was recommended prior to the results of the RAPIDO-trial were known. After that TNT as in RAPIDO or according to the LARCT-US protocol is the preferred schedule. If the patient cannot tolerate CRT/TNT, scRT with delay is recommended.

W&W is practised primarily if a cCR is obtained. If intentional, CRT is the preferred treatment. Participation in a trial is recommended.

a If the extent of peritoneal involvement is limited, direct surgery is recommended.

b If the MRF-involvement (<1 mm) is against an easily resectable organ/structure, 5 × 5 Gy is recommended.

c 4 or more nodes having at least 2 of the 3 criteria, size above 5 mm, irregular border, and heterogeneous appearance.

In the most recent generation of trials, all claiming to have restricted inclusion to patients with LARC only, great variability in what stages were actually included are seen. As shown in Table 2, the proportion of patients with stage cT4 has varied from 0 to about 30% except in a Polish trial reaching just above 60% (52). After the RAPIDO trial had closed patient entry in June 2016, 462 LARC patients with risk factors for recurrence from Sweden were, during a 3-year period, treated within a pragmatic phase II trial (LARCT-US, NCT03729687) or outside the trial in real-life; 52% and 60% of the patients had stage cT4 and 72 and 80% had MRF-positivity (Glimelius, unpublished information. No outcome data are yet available).

**Table 2:** Overview of chiefly randomised studies providing total neo-adjuvant therapy (TNT) or neoadjuvant chemotherapy without radiotherapy to patients with locally advanced rectal cancer.

align="left">Trial/study (ref)	align="center">Type of study	align="center">Treatment	align="center">Number of patients	align="center">cT4 (%)	align="center">MRF+ (%)	align="center">pCR^a^	align="center">CR^b^	align="center">LRR 3–5 years (%)	align="center">DM 3–5 years (%)	align="center">Comments
**Randomised phase III trials comparing TNT vs CRT (±AC)**										
Polish (53, 83)	Rand phase III	TNT scRT+FOLFOX x2	261	63	NR	17		7	35	Early difference in OS, not DFS, after 8 years, no difference. Locoregional control, 66% versus 68%.
		CRT	254	64	NR	12		5	33	
RAPIDO (33, 84)	Rand phase III	TNT, scRT+CAPOX x6	460	32	62	28	29	9	20	DrTF (primary endpoint): 3 years 24% versus 30%, 5 years 28% versus 34%
		CRT, optional AC	446	30	60	14	14	5	27	
Stellar (85)	Rand phase III	TNT, scRT+CAPOX x4+AC	302	16	56	17	22	7	22	Non-inferiority trial, DFS no difference (non-inferior) 12% versus 6% refused surgery
		CRT +AC	297	13	56	12	12	8	23	
PRODIGE-23 (34)	Rand phase III	TNT, FOLFIRINOXx6+CRT +AC	231	18	26	28	26	4	17	DFS (primary endpoint) 3 years 76% versus 69%
		CRT + AC	230	16	23	12	11	6	25	
**Randomised phase II trials comparing TNT vs CRT (±AC)**										
Spanish GCR-3 (86)	Rand phase II	TNT, 4 CAPOX+ CRT	56	13	0	14		5	25	No benefit seen, better compliance to chemotherapy
		CRT+AC	52	6	10	13		2	21	
INOVA (87)	Rand phase II	TNT, 2 FOLFOX+bev + CRT	46	0	NR	24		NR	NR	Adding bev. to CRT did not improve pCR
		CRT with bev	45	0	NR	11		NR	NR	
WAIT (88)	Rand phase II	CRT+ 3 FLv dG +AC	25	4	60	16		NR	NR	No benefit of 3 FLv dG consolidation
		CRT+AC	24	20	50	25		NR	NR	
KCSG CO 14-03 (89)	Rand phase II	CRT+2 CAPOX+AC	53	17	26	14		NR	NR	Marginal benefit of 2 CAPOX consolidation
		CRT+AC	55	18	29	6		NR	NR	
Marechal (90)	Rand phase II	2 FOLFOX+CRT	29	7	NR	25		NR	NR	No benefit of 2 induction FOLFOX
		CRT	28	10	NR	28		NR	NR	
**Randomised phase III trials comparing chemotherapy and selective CRT vs CRT**										
GRECCAR 4 (91)	Rand phase II	TNT, induction FOLFIRINOX, good responders, surgery, or CRT	30	0	NR		10% versus 58%	0	15	Good responders randomised to surgery or CRT
		Poor responders, CRT 50 or 60 Gy	103	24	NR		17	5	18	Poor responders randomised to CRT 50 or 60 Gy, no difference
PROSPECT (92)	Rand phase III	TNT, FOLFOX+ selective CRT	597	0	NR	22		2	NR	FLOFOX non-inferior to CRT concerning DFS (primary endpoint), 81% versus 79%. 9% received CRT in early/intermediate tumours
		CRT	597	0	NR	24		2	NR	
**Randomised phase III trials comparing different TNT schedules**										
AIO-12 (93, 94)	Rand phase III	TNT induction 3 FOLFOX +CRT	156	18	NR	17		NR	NR	DFS 3 years 73% both arms. Consolidation FOLFOX resulted in slightly higher pCR. Probably no long-term benefit. pCR not higher than CRT in earlier trial (95)
		TNT CRT+consolidation FOLFOX	150	12	NR	25		NR	NR	
FOWARC (96, 97)	Rand phase III	TNT, FLv dGx3 +CRT + AC dG	165	35	NR	14		8	NR	DFS (primary endpoint) 3 years 73, 77 and 74%. More pCR with oxaliplatin but no other benefit. Chemotherapy alone resulted in fewer pCR
		TNT, mFOLFOXx3+CRT + AC mFOLFOX	165	34	NR	28		7	NR	
		mFOLFOX6 x4-6 +AC mFOLFOX	165	30	NR	7		8	NR	
OPRA (75, 98)	Rand phase II	Induction FOLFOXx8/CAPOXx5 +CRT	158	15	NR		50	6	16	Worse TME-free survival with induction (41% versus 53%). More regrowth. DFS 3 years similar 76%. A near-CR means more regrowth but still chance for W&W
		CRT+consolidation FOLFOXx8/CAPOXx5	166	11	NR		67	6	18	
GEMCAD 1402 (99)	Rand phase II	TNT, FOLFOXx6+aflibercept + CRT	115	18	59	25		5	17	More pCR if + aflibercept, no other difference.
		TNT, FOLFOXx6 + CRT	65	20	57	15		6	17	
PANEX (100)	Phase II × 2	TNT, CAPOXx4 + CRT + AC	269	22	62	20		6	21	Randomised ±cetuximab, no difference, pooled data presented
**Phase II studies, other designs including propensity score matching**										
Garcia-Aguilar (101, 102)		CRT +AC	60	2	NR	18		NR	NR	Follow-up reported later, however, many missing, slightly different results. Claiming improved DFS if consolidation chemotherapy
		CRT+FOLFOXx2	67	1	NR	25		NR	NR	
		CRT+FOLFOXx4	67	4	NR	30		NR	NR	
		CRT+FOLFOXx6	65	5	NR	38		NR	NR	
Ng (103)	Phase II	FOLFOXx3 interdigitating CRT with oxaliplatin	40	12	NR	20		5	23	
Grabenbauer (80)	Propensity score matched	CRT	114	15		16		4	22	More pCR with + oxaliplatin, no other difference
		CRT with oxaliplatin	114	15		27		4	21	
Engels (104)	Rand phase II	RT+integrated boost	82	11	34	14		7	23	More pCR with CRT, no other differences
		CRT	86	6	36	24		6	26	
Yamaguchi (105)	Retrospective propensity score	TNT, induction chemo +CRT	130	22	NR	26		2	NR	DFS 84% versus 71%
		CRT+AC	130	25	NR	10		6	NR	
AVACROSS (106)	Phase II	TNT, CAPOX+bev +CRT	47	12	19	36		2	16	Short follow-up, mean 32 months
Benlice (107)	Retrospective propensity score	TNT, scRT+ FOLFOXx4-6/CAPOXx3	53	27	NR	21		NR	NR	48 scRT+chemotherapy matched with 48 CRT+chemotherapy patients with similar pCR rates, 21% versus 19%.
		TNT CRT+ FOLFOXx4-6/CAPOXx3	128	17	NR	20		NR	NR	
		CRT	164	25	NR	15		NR	NR	
Moyer (108)	Retrospective	TNT FOLFOXx8/CAPOXx5+CRT	84	19	NR		43	NR	NR	Compared two different hospitals using different treatments
		TNT scRT+FOLFOXx8/CAPOXx5	83	27	NR		53	NR	NR	
PROARCT (103)	Phase II	TNT, FOLFOX+ split RT	40	12	NR	20		3	28	
Chin (109)	Phase II	TNT, scRT+ optional boost RT +FOLFOX x8	90	20	42		50	NR	NR	TME-free survival at 2 years 47%, local regrowth in 21%
Hall (110)	Phase II	TNT, FOLFOX+CRT	121	29	NR	28		NR	NR	Evaluated MRI for pCR prediction
COPERNICUS (111)	Phase II	FOLFOXx4+scRT+AC	60	5	0	12		4	14	Short follow-up
Myerson (112)	Phase II	TNT, scRT + FOLFOXx4	76	9	NR	25		5	7	
Markovina (113)	Retrospective, matched	TNT, scRT + FOLFOXx4	69	7	NR	28		8	12	
		CRT	69	7	NR	16		4	30	
Kim MSKCC (114)	Retrospective	TNT induction FOLFOX/CAPOX+CRT	313	13	NR		27	NR	NR	No benefit besides more CR (pCR+cCR). DFS, LRR and DM curves presented, no difference
		CRT+AC	311	6	NR		20	NR	NR	
KIR (115)	Rand Phase II	FOLFOXx6+HDBRT +AC	120	0	32	31		6	20	No benefit of induction FOLFOX
		HDBRT +AC	60	0	25	28		6	20	
Goffredo (116)	Register study	TNT	3076	NR	15	14		NR	NR	US National Cancer Database study 2006–2015, limited information
		CRT	5472	NR	17	12		NR	NR	
Cercek (117)	Retrospective	TNT, 4 month chemotherapy+ CRT	308	6	NR		36	NR	NR	Material from a comprehensive cancer centre. Low cT-stage associated with CR
		CRT +AC	320	12	NR		21	NR	NR	
**Phase II studies delivering neoadjuvant chemotherapy alone**										
GEMCAD 0801 (118)	Phase II	4 CAPOX+bev + AC	46	2	0	20		5	17	
Uehara (119)	Phase II	CAPOX+bev	32	59	50	13		10	21	Japanese series, more advanced tumours than in most Western world studies
Ishii (120)	Phase II	IFLx2	26	12	NR	4		NR	NR	Japanese series
Mukai (121)	Phase II	CAPOX ± bev + AC	61	38	NR	8		NR	NR	Japanese series
Matsuda (122)	Phase II	CAPOXIRI	55	29	NR	8		4	NR	Japanese series

NR, not reported, TNT, total neoadjuvant therapy, CRT, chemoradiotherapy to about 45–50 Gy with a fluoropyrimidine, scRT, short-course radiotherapy to 5 × 5 Gy in 1 week, HDBRT, high-dose brachy radiotherapy, AC, adjuvant chemotherapy, bev, bevacizumab, chemo, chemotherapy, FLv, 5-FU/leucovorin; dG, de Gramont schedule of 5FU/leucovorin; CAPOX, capecitabine + oxaliplatin; FLOFOX, 5FR/leucovorin + oxaliplatin; FOLFIRINOX, 5FU/leucovorin + irinotecan + oxaliplatin, IFL, irinotecan, bolus 5FU, leucovorin.

a pCR invariably evaluated in resected patients only in per protocol patients or in an intention-to-treat population. Thus, it is not possible to make direct comparisons between trials. CR generally presented in the intention-to-treat population. If rate of pCR is presented, CR is usually not presented, or the reverse. If both are presented, as in the phase III trials, W&W was not an option according to the protocol but practised in a few patients.

b pCR or cCR, that is entered W&W, no regrowth within approximately 1 year.

## Timing of surgery, organ-preservation or watch-and-wait (W&W)

A Brazilian group reported in 2004 that CRT could result in a complete clinical remission (cCR) in preferably early and small tumours, not primarily needing any pre-treatment for excellent tumour control, and that surgery could be postponed indefinitely, that is organ preservation (11). This concept has been gradually accepted in the Western world. A W&W strategy can be applied in two different situations, ‘intentionally’ or ‘if-it-happens’. A pCR of pre-treated rectal tumours at surgery was seen not infrequently, and, in the light of the favourable experience from Brazil, many surgeons increasingly questioned the necessity of surgery and delayed surgery if an excellent response was seen after RT/CRT. The recurrence risk in patients with pCR is low (25), and the same is the case if cCR is reached (54). After pioneering work at a few hospitals with special interest and the development of strict criteria for the evaluation of cCR and follow-up routines, this has now been widely accepted and practised worldwide. International registration is performed (International Watch & Wait database, IWWD) and regularly updated (55–58). With many thousands of patients presently registered from multiple centres, it can be concluded that outcomes in those who respond with cCR at an evaluation about 12 weeks after RT/CRT are favourable. Regrowth rates in the bowel are approximately 25%, but most of the regrowth can be salvaged by subsequent surgery and few get either a local or systemic recurrence (59–61). The W&W strategy requires careful evaluation of the response and regular follow-up, including palpation, endoscopy and pelvic MRI. International consensus recommendations have been developed (62).

A W&W strategy in patients where the tumour happens to respond completely is presently accepted worldwide. The chance to obtain a cCR (or a pCR if the tumour is resected) varies considerably. Smaller and thus earlier stage tumours respond more often. Besides stage/tumour length/size, multiple factors have been associated with pCR/cCR, but none have consistently been associated aside from a non-elevated serum carcino-embryonic antigen (CEA) level before treatment, indicating an approximately doubled chance of response (63, 64). There is a belief that longer waiting times than usual result in more pCR, as indicated by multiple retrospective studies (65–68), but this has been difficult to prove in randomised studies (69–73). In patients where it is believed to be advantageous to wait since a decrease in the tumour has been observed at an initial evaluation, often designated near complete response or nCR, you will, for obvious reasons, see not only more pCR/tumour regressions but also fewer recurrences since responding patients do better. The delay in itself cannot improve outcome unless it is accompanied by active treatment. A nCR at initial evaluation may, in many patients, develop into a cCR and result in prolonged organ preservation. The risk of local regrowth is, however, higher (about 40–50% rather than 20–30%) as is the risk of distant metastases (74, 75).

Intentional W&W, that is to give CRT to patients where the need for any pre-treatment to lower risks of recurrences is otherwise not present, is practised at many hospitals worldwide. The wishes to avoid major surgery, that is not to have a stoma after an abdominoperineal excision (APE) or a disabling low anterior resection syndrome (LARS), may be many. Not only the best candidates are those with small early tumours, but also large bulky tumours may respond (76, 77). They should, based on present knowledge, obtain a combination of RT and chemotherapy, such as standard CRT (most patients have so far been treated with this) or a more intensified scheduled with higher radiation doses, addition of local RT [e.g. using brachytherapy (78) or contact therapy as in the OPERA trial (79)], more intense chemotherapy than a fluoropyrimidine alone concomitant with the RT [although this has not markedly increased pCR-rates albeit seen in a meta-analysis (80), operated with a transanal local procedure rather than TME (60) or providing TNT (to be discussed below)]. In early stage tumours, chemotherapy alone followed by a local procedure has also resulted in favourable outcomes in small series (81). Properly selected, up to every other patient can then obtain a cCR, but, still, most patients do not respond, and, thus, toxicity from both RT/CRT and surgery and regrowth is not infrequent (6). Ongoing trials such as Star-Trec trial (NCT02945566) or NOMINATE (82) may help to find the appropriate patients/tumours and identify better treatments.

## Total neoadjuvant treatment

Due to the lack of clear DFS and OS benefits despite excellent locoregional control from more efficient RT/CRT and better surgery and the lack of sufficient benefit from adjuvant chemotherapy, recent interest has focused on providing the systemic treatment before surgery, that is neoadjuvant. The systemic treatment may, thus, potentially kill more tumour cell deposits that could have grown during the waiting time and/or promoted by the surgery. Furthermore, compliance to postoperative chemotherapy after rectal cancer surgery has been poor. Randomised trials have now shown that compliance to the treatment has become better, more tumours have disappeared (increased pCR rates) and fewer systemic recurrences are seen (Table 2).

Of greatest relevance are the randomised phase III studies comparing TNT with the reference treatment, CRT (33, 34, 53, 85) and also randomised phase II studies with the same comparator (86–90). With the exception of the Polish study providing chemotherapy for only 1 month, the other phase III studies showed that TNT gives more pCR. Two of them showed fewer distant metastases and, thus, improved DFS/DrTF, but none showed any OS benefit. The lack of an OS benefit has been stressed, providing a word of caution (1, 123).

The studies have had different inclusion criteria, and the TNT schedules have varied. For these reasons, it is difficult to draw conclusions about which patients benefit the most and what the most effective schedule is. Nevertheless, it is my belief that TNT is the right way forward. The weakest part is the systemic chemotherapy; it does not have sufficient capability to eradicate the subclinical disease that is present in many patients. Even if many are impressed by the effects of systemic treatments in metastatic disease where initially non-resectable tumours can be resected for cure after downsizing, the cell kill effect is limited and some tumours do not respond having progressive disease as best response. Consequently, it is attractive to provide RT first resulting in several logs of cell-kill in most tumours within the irradiated volume. It should be provided as soon as possible not delaying the initiation of effective chemotherapy. In this respect, scRT for 1 week with possibilities to initiate the systemic treatment about 2 weeks later is desirable. Between 5 and 8% of the patients experience grade 3 toxicity to scRT, delaying the start of chemotherapy (32, 33, 40, 124). It is also my belief that the chemotherapy after the radiation should not be too long since some tumours will not respond well or actually progress during the chemotherapy. Although not proven, the six cycles of CAPOX in RAPIDO may be needed to prevent distant metastases in many patients, but some tumours will progress during the treatment. Four cycles as in STELLAR and used in a Swedish phase II/real-life study LARCT-US (NCT03729687, results to be published) may, in this respect, be more optimal, but the STELLAR trial did not show any decrease in distant metastases. However, no increased LRR rates as reported after 5 years in RAPIDO (84, 85) were seen.

TNT, either using scRT + chemotherapy or CRT with induction or consolidation chemotherapy, does not increase postoperative morbidity relative to CRT (33, 85, 124–126). An increased risk of a breached mesorectum in the RAPIDO TNT arm may be an indication of greater surgical difficulties, similar to what was noted in one trial exploring the value of prolonging the interval to surgery after CRT (69).

TNT for some patients with LARC is presently on the right track for improved therapy, but uncertainties remain. It will not be tolerable among many old and/or frail patients. It will definitely not be needed for large groups of patients with LARC; most trials until now have included all tumours considered to be LARC (cT3–4 or cN+, see above) or have excluded the most advanced/ugly ones. The proportion of cT4 has varied from 0% to above 50% reflecting the different inclusion criteria (Table 2).

Several subsequent trials are ongoing, and results already provide clues to some uncertainties (see Table 2). The best RT is not known (scRT or CRT? dose escalated RT?). Concerns have been expressed regarding the use of scRT after the increased risk of LRR seen in RAPIDO (84, 127). CRT and scRT with immediate surgery have, as described earlier, been compared in two randomised trials with no difference in efficacy, but none have compared CRT with scRT and delayed surgery. Retrospective studies indicate that pCR is seen less frequently after scRT than after CRT (128), indicating that the cell kill effect may be less. Using scRT, ‘full-dose’ systemic treatment can be initiated after 3–4 weeks, whereas it requires at least 8–10 weeks after CRT. The concomitant fluoropyrimidine, sometimes combined with other drugs, used for radiosensitising in CRT is not without systemic effects albeit not as effective as when given in full doses without the radiation.

Nor is it known if chemotherapy should be provided after radiation (consolidation) or before (induction). At least two trials indicate that CRT first results in better oncological outcome (93, 94, 98). It is not known whether triple chemotherapy, as used in PRODIGE-23 (34) or GRECCAR-4 (91) or a doublet as used in the other trials, is advantageous. Most probably, the duration cannot be too long since local progression may occur in some patients even if longer treatment could kill subclinical disease in other patients. In order to reach pCR-rates above 20% (not reached using CRT in LARC) or CR-rates above 40–50%, it may be needed to deliver 3–4 months of chemotherapy after scRT (33, 85, 107, 112, 113), whereas 1–2 months is not (53, 88–90, 111).

Two trials, PROSPECT (92) and GRECCAR-4 (91), asked the question as to whether CRT could be omitted if the response to induction chemotherapy is good. In the PROSPECT trial, FOLFOX induced at least a 20% decrease in tumour size in over 90% of the patients, and these patients proceeded directly to surgery, whereas those with less response had CRT before surgery. The results in these patients were non-inferior to those randomised to CRT alone. This study did not include any cT4-tumours, which could explain the favourable results. The GRECCAR-4 study first treated the patients with triple chemotherapy, FOLFIRINOX and if good response, reached in 30/133 patients, randomised the patients to surgery directly or CRT followed by surgery. These responding patients, where none had cT4-stage fared well, whereas most patients who did not respond fared worse after CRT using either 50 Gy or dose-escalated 60 Gy. It is, thus, possible to select a group of patients whose tumours are sensitive to chemotherapy and omit CRT with its late toxicity. This may constitute an argument for starting with chemotherapy although this sequence appeared worse in two trials (93, 94, 98).

Substantial progress in the treatment of LARC can only be reached with more efficient systemic treatments. Even after delivery of the most efficient systemic treatment available today in CRC and using the sequence considered the best, the advances have been only minor. Available drugs with reasonable efficacy in mCRC have been around for 10–15 years, some for much longer. Although we can handle them better, particularly in conjunction with oncosurgery, the cell kill effect is insufficient. Survival for treatable mCRC patients with favourable tumour characteristics has been prolonged (129), but for the population, improvements are a few months at the best (130). An exception relates to the marked antitumour effects of checkpoint inhibitors in microsatellite instable (MSI) CRC. Overall, about 15–20% of CRC are MSI, but most of them are right-sided. In rectal cancer, only a few per cent are MSI. However, in small patient series, remarkable effects have been noted in LARC with complete disappearance clinically or at surgery (131–133). Other recently developed drugs against KRAS G12C mutated tumours could also potentially be included in the armamentarium in primary rectal cancer (134, 135). This progress cannot solely depend upon research in rectal cancer but must rely on general oncologic pre-clinical and clinical research.

## Conclusions

In patients with a rectal cancer sufficiently advanced to require neoadjuvant treatment to improve outcome, it appears motivated to defer surgery if the tumour responds with a complete remission and includes them in a W&W programme. The best treatment for this purpose is not known, but it does not appear to be important which treatment has been used if cCR is reached. The first evaluation should be performed reasonably early (5–6 weeks), so that surgery is not delayed unnecessarily if the tumour has not responded well. In non-sufficiently responding tumours, further delay is without benefit and may be deleterious. If a near-complete response is seen, further delay of another 6–7 weeks appears safe, and W&W could be initiated if cCR (or sufficient response for local surgery/boost RT) is seen. The initiation of intentional organ-preservation in early/intermediate risk tumours must be based upon a discussion between the patient and the doctor where pros and cons are appropriately considered.

In patients with tumours at high risk of recurrence, particularly systemic recurrences, TNT appears to be a logical step forward. It will decrease the risk of distant metastasis and, thus, has the potential to cure more patients. The benefits have been shown in randomised trials, but they are not marked. The best patients/tumours for this treatment are not known, but it should at least not be applied to all patients considered to have a ‘locally advanced rectal cancer’. This would mean over-treatment of many patients. MRI-based N-positivity, not even N2-positivity, is not a reliable criterion to select for TNT. Much remains to optimise and find the best schedule although the greatest need of improvement relates to the systemic treatment.
